# Copper sulfide deposition and remobilisation triggered by non-magmatic fluid incursion in the single-intrusion Tongchang porphyry system, SE China

**DOI:** 10.1038/s41598-024-52978-5

**Published:** 2024-01-31

**Authors:** Xuan Liu, Antonin Richard, Jacques Pironon, Kuifeng Yang

**Affiliations:** 1 Geological Survey of Finland, P.O. Box 96, 02151 Espoo, Finland; 2 Université de Lorraine, CNRS, GeoRessources, 54506 Vandoeuvre-lès-Nancy, France; 3 Key Laboratory of Mineral Resources, Institute of Geology and Geophysics, Chinese Academy of Sciences, Beijing, 100029 China; 4 Institutions of Earth Science, Chinese Academy of Sciences, Beijing, 100029 China

**Keywords:** Geochemistry, Economic geology

## Abstract

Porphyry ore deposits are a major source of base and precious metals. Likewise, they bear important fingerprints for understanding magmatic / hydrothermal processes in the convergent margin. For many decades, the sources of non-magmatic fluid and its role in sulfide mineralization in the porphyry hydrothermal systems have been equivocal. The Tongchang porphyry deposit, which is a single intrusive system with a well-established fluid history, is investigated to reconstruct its hydrothermal process that contributed to the ore formation. In-situ oxygen and strontium isotopes in hydrothermal quartz and anhydrite revealed a coexistence of magmatic and non-magmatic fluid reservoirs. The granodiorite—derived magmatic fluid and external groundwater were spatially separated by a hydrologically impermeable shell formed by retrograde mineral deposition (mainly quartz). The location of the impermeable shell coincided with a brittle-ductile transition (BDT) interface established in the host phyllite in response to latent heating by the cooling magmas. It is inferred that the ductile phyllite beneath the impermeable shell may have entrained some amounts of groundwater and remnant metamorphic fluid. The early fluid stage was dominated by the magmatic fluids, forming disseminated chalcopyrite and barren quartz veins in the potassic-altered ductile granodiorite at high temperatures (> 500 °C). The next stage (early-intermediate) was also driven by the circulation of the magmatic fluids, but in a largely brittle zone formed in-between the impermeable shell and the retreated BDT interface (similar to the so-called “carapace” in the orthomagmatic models). In this stage the formation of pyrite and chalcopyrite veins together with chloritic alteration at temperatures of 400–350 °C occurred. The late-intermediate stage was marked by incursion of the trapped non-magmatic fluids due to rupturing of the enlarged carapace. Mixing of the non-magmatic fluids and the magmatic fluids led to deposition of a major phase of vein-type Cu sulfide at temperatures of 350–300 °C. The late fluid stage was characterized by breaching of the impermeable shell in response to volumetric contraction of the fluid system, leading to excessive infiltration of groundwater and ore remobilization. Based on the Tongchang model, six generic fluid models are proposed for porphyry ore deposits that differ in availability of non-magmatic components as well as intrusive histories. The models can account for variabilities in ore and alteration styles found in porphyry ore deposits globally.

## Introduction

Magmatic hydrothermal processes are the most significant vector for mass and heat transfer in the crust^1^. The emplacement and interaction of hydrous magmas with surrounding rocks and fluid reservoirs dictates the formation of mineral and energy resources in porphyry and geothermal systems^2–6^.

For porphyry ore deposits, the world’s major sink of Cu, Mo, Au, Ag, and Re^6^, orthomagmatic models have been widely adopted which envisage magma as the dominant source of heat and materials (fluid, metal, and ligand)^7–11^. The models commonly support the notion that copper deposition takes place in the early stage in response to fluid cooling, decompression, and fluid/rock interaction^12,13^. External non-magmatic waters (groundwater, connate water, metamorphic water, etc.), otherwise, function merely as coolant for the magmas and magmatic fluids^4,6^, and are only significant for the late, ore—barren processes^14,15^. However, several H–O isotope studies have discovered non-magmatic δD and δ^18^O signatures in the early potassic stages^16–20^. The non-magmatic incursion has been considered as an enabler for sulfide deposition^2,13,19^. For instance, a recent secondary ion mass spectrometry (SIMS) oxygen isotope study demonstrated that Cu sulfides in the Bingham Canyon porphyry ore veins precipitated from a hybrid fluid containing appreciable amounts of meteoric waters^19^.

The longstanding debate surrounding non-magmatic incursion is largely due to two common geologic complexities in the porphyry deposits. The first one is cyclicity of fluid flow induced by multiple generations of intrusion^21,22^, which may lead to a poorly constrained fluid flow sequence. The fluid flow sequence might be further complicated by fluid overprinting and resulting complex quartz microtextures as documented by scanning electron microscopy—cathodoluminescence (SEM-CL) studies^12,23–26^. Mono-intrusion porphyry systems are of particular interest because they may have simpler thermal and fluid history compared to multi-intrusion ones^21,22^, therefore for this study the Tongchang mono-intrusion porphyry deposit was selected^26^.

Previous O–Sr isotopic studies of this deposit utilized bulk-rock method and, not unexpectedly, ended up with incompatible conclusions. Spatial O isotope analyses of altered rocks revealed ^18^O enrichment (δ^18^O = 12‰) in shallow levels yet^18^O depletion at depths (δ^18^O = 6.8‰) compared to the unaltered porphyry (δ^18^O = 8.4‰), suggesting a main role of non-magmatic fluids^27,28^. By contrast, oxygen isotope analyses of quartz and phyllosilicate minerals revealed typical magmatic δ^18^O signatures, and thus supported orthomagmatic models^29,30^. This discrepancy, common in porphyry deposits, is primarily due to sub-mineral isotopic heterogeneity that can barely be detected by bulk analysis^18^. Moreover, O isotopes redistribute easily during fluid–rock interaction and thus are not a fully reliable indicator of fluid source^14–16^. Strontium isotopes, on the other hand, are more resistant to redistribution during water–rock interaction^31,32^ and have been successfully used for fluid tracing^31,33^. Therefore, this study combines in-situ SIMS O isotope analyses of quartz and laser ablation multi-collector inductively coupled mass spectrometry (LA-MC-ICP-MS) Sr isotope analyses of anhydrite for the Tongchang porphyry Cu deposit with the main objective of detecting potential non-magmatic components and understanding their role in the deposit genesis.

## The Tongchang deposit

**Figure 1:**
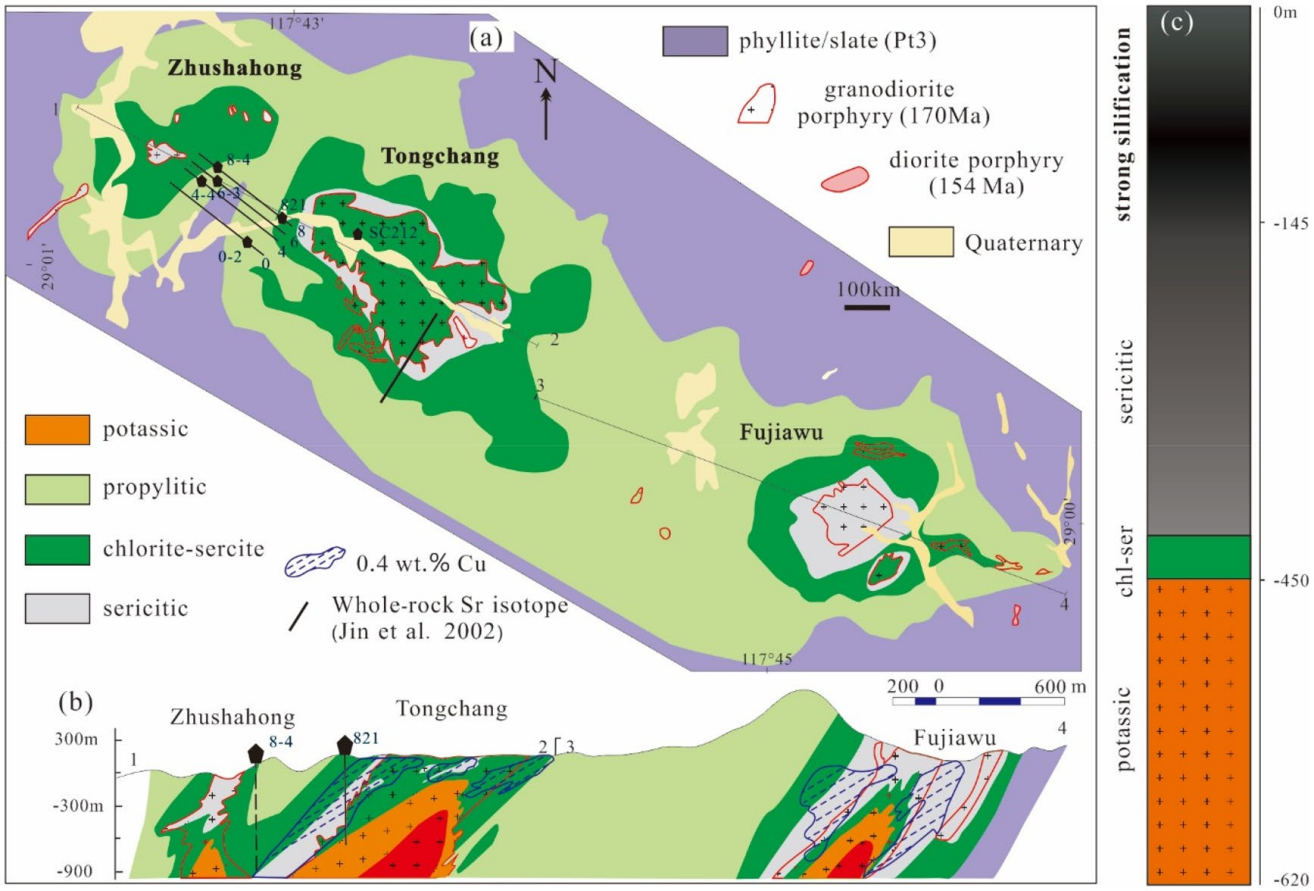
(**a**) A geological map of the Dexing porphyry Cu district showing distribution of granodiorite (zircon U–Pb age ca. 170 Ma) and diorite porphyries (zircon U–Pb age ca. 154 Ma) intruding phyllite and slate (Pt3, Neoproterozoic) along with potassic, propylitic, chlorite-sericite, and sericitic alteration zones (adapted from Liu et al.^26^); (**b**) A cross section profile of the ore district showing vertical distribution of rocks, alterations, and ores (adapted from Liu et al.^26^). Locations of samples examined in this study and for whole-rock Sr isotope study by Jin et al.^27^ are also shown; (**c**) A simplified column showing strong silicification in the phyllite and slate at shallow depths in drill core ZK821. The figure was reproduced using CorelDRAW2019 (http://www.coreldraw.com/en/).

The Tongchang porphyry Cu deposit along with the Fujiawu and Zhushahong deposits are located in a Neoproterozoic orogen that sutured the Yangtze and Cathaysia blocks^34^. The three deposits, unlike most other porphyry deposits around the world, are associated with a single granodiorite porphyry intrusion^35^ (Fig. 1a). The three granodioritic intrusions, intruded into the Neoproterozoic Shuangqiaoshan Group (mainly phyllite and tuffaceous slate), emanated from a common batholith at depth, derived from re-melting of subduction-modified lithosphere at ca. 170 Ma^34,36^ (Fig. 1a). Over 95 vol.% of the magmatic rocks discovered at Dexing are granodiorite porphyries, with minor amounts of late diorite porphyry and aplite (U–Pb age ca. 154 Ma^37^).

Hydrothermal alteration and veins are comparable to the typical porphyry Cu deposits^5^. Potassic and propylitic alterations were developed in the granodiorite and wallrock (mainly phyllite), respectively, defining concentric zones around the granodiorites. These early-formed alterations were overprinted first by chlorite-sericite and then by sericitic alterations along granodiorite/wallrock boundaries and fractures/faults (Fig. 1b). Silicic alteration (silicification) has been documented in previous works^38^, but no discrete zones have been distinguished. Our field and petrography observation suggest that there are wide zones of silicification overprinting the propylitic alteration in the shallow levels and grading into the sericitic alteration at depths (Fig. 1c). Systematic mapping is needed to further constrain the extent and shape of the silicic zone.

Hydrothermal vein types and classification have been detailed by Liu et al.^26^. Five A-type veins, three B-type veins, and five D-type veins are analyzed in this study (Table 1), including hematite-quartz veins (abbreviated as A.V_hq_, “A” denotes A-type, “h” and “q” the subscripts represent hematite and quartz, arranged in an increasing volume), K-feldspar-quartz veins (A.V_kq_), anhydrite-quartz veins (A.V_aq_), chalcopyrite-pyrite-quartz veins (B.V_cpq_), molybdenite-bearing anhydrite-chalcopyrite-pyrite-quartz veins (B.V_macpq_), molybdenite-bearing quartz-pyrite veins (D.V_mqp_), and pyrite-dominant veins (D.V_pd_). Minerology and crosscutting relation suggest that A.V_hq_, A.V_kq_, A.V_aq_ are broadly comparable to the so-called A-type veins, B.V_cpq_, B.V_macpq_, to the B-type veins, and D.V_mqp_ and D.V_pd_. to the D-type veins. Copper minerals, mainly chalcopyrite and subordinately bornite and Cu-sulfosalts, are localized in the chlorite—sericite and sericitic zones (Fig. 1b). In the chlorite-sericite zone, chalcopyrite occurs in veinlets (B.V_cpq_ and B.V_macpq_) and disseminations in the altered host rocks. In the sericitic alteration zones, chalcopyrite occurs as mineral inclusions, and tennantite as disseminations in the altered rocks^39^.

**Table 1:** A summary of sampling locality and petrography of the analyzed rock samples of the Tongchang porphyry Cu deposit.

Sample no.	Sampling location	Vein type	Host rock alteration
12LDX37	Drill hole SC212 171 m	A.V_hq_	The rock is significantly affected by chlorite-sericite alteration. Primary mafic minerals are replaced by chunks of chlorite, sericite, hematite, muscovite, and chalcopyrite. Feldspar phenocrysts are replaced by sericite. Quartz phenocrysts remain largely unaffected. Groundmass is replaced by fine-grained hematite and chalcopyrite. The rock is intensively crosscut by hematite quartz veins (V_hq_), which are subsequently cut by chalcopyrite pyrite quartz veins and veinlets (V_cpq_)
10DX172	Drill hole ZK8-4 933 m	A.V_kq_	Plagioclase phenocrysts are partly (less than 50% of the surface) altered by sericite along cleavages and cracks. K-feldspar phenocrysts are completely replaced by sericite. Unidentified mineral pseudomorphs of inner sericite and outer chlorite, hematite and magnetite are present. Primary mafic minerals are replaced by chunks of small biotite, chlorite, hematite, and magnetite. Groundmass consists of sericite, quartz, biotite, hematite, and magnetite
12DXF05	Open pit 230 m platform		Feldspar phenocrysts are overgrown by K-feldspar, which are partly altered by sericite. Primary biotites are replaced by quartz, sericite, chalcopyrite, pyrite, and rutile. Quartz phenocrysts remain largely unaffected. Numerous chunks of chlorite are present. Groundmass consists of fine-grained quartz
10DX168	Drill hole ZK8-4 943 m	A.V_aq_	K-feldspar phenocrysts are pseudomorphed by sericite, and some anhedral K-feldspar grains are partly altered by sericite, chlorite, and epidote. Clusters of chlorites, sericite, anhydrite, epidote and molybdenite are present. Some quartz phenocrysts are overgrown, and others remain unaffected
12DX500	Drill hole ZK821 620 m		K-feldspar phenocrysts are largely replaced by sericite, and plagioclase is slightly altered by sericite. Primary biotite remains largely unaffected, and some biotites are related by small, unoriented biotite, which are replaced by chlorite, sericite, quartz, hematite, and magnetite. Groundmass consists of quartz, sericite, and chlorite
10DX22	Drill hole ZK8-4 587 m	B.V_cpq_	The host rock is a diorite porphyry. Feldspars are completely replaced by sericite in the center and chlorite in the rim. Groundmass mainly consists of small biotite, sericite, and minor amounts of quartz
12DX328	Drill hole ZK821 499 m	B.V_macpq_	The center of feldspar phenocryst is replaced by chlorite while rim is replaced by sericite. Primary mafic minerals are replaced by chlorite, rutile, chalcopyrite, hematite, and magnetite. Aggregations of chlorite-rutile- magnetite, chlorite-chalcopyrite-rutile-anhydrite are present. Groundmass consists of quartz, anhydrite, rutile, and muscovite. Molybdenite occurs in both alteration and vein
10DX164	Drill hole ZK8-4 952 m	D.V_mqp_	Feldspar phenocrysts are pseudomorphed by sericite. Mafic mineral pseudomorphs are not common. Where observed, they consist of chlorite and pyrite. The rock is intensely silicified. Quartz in the groundmass is medium-sized, intergrowing with abundant muscovite and chlorite. Sulfides are dominated by pyrite and occur as aggregations and bands
10DX220	Drill hole ZK8-4 601 m		The immediate host rock consists of strong Sericitic alteration halos of the molybdenite quartz pyrite vein. In the halo, primary minerals except quartz are completely replaced by large muscovite, rutile, quartz, and pyrite. No mineral pseudomorphs are eliminated. Groundmass consists of fine-grained quartz and large muscovite
10DX140	Drill hole ZK0-2 337 m	D.V_pd_	The rock is a sericitic altered phyllite, consisting of oriented sericite, quartz, pyrite, and rutile
10DX145	Drill hole ZK4-4 410 m		It is a sericitic altered phyllite, consisting of oriented sericite, quartz, pyrite, and rutile
10DX201	Drill hole ZK8-4 738 m		It is a breccia consisting of pyrite, quartz, and carbonate

**Figure 2:**
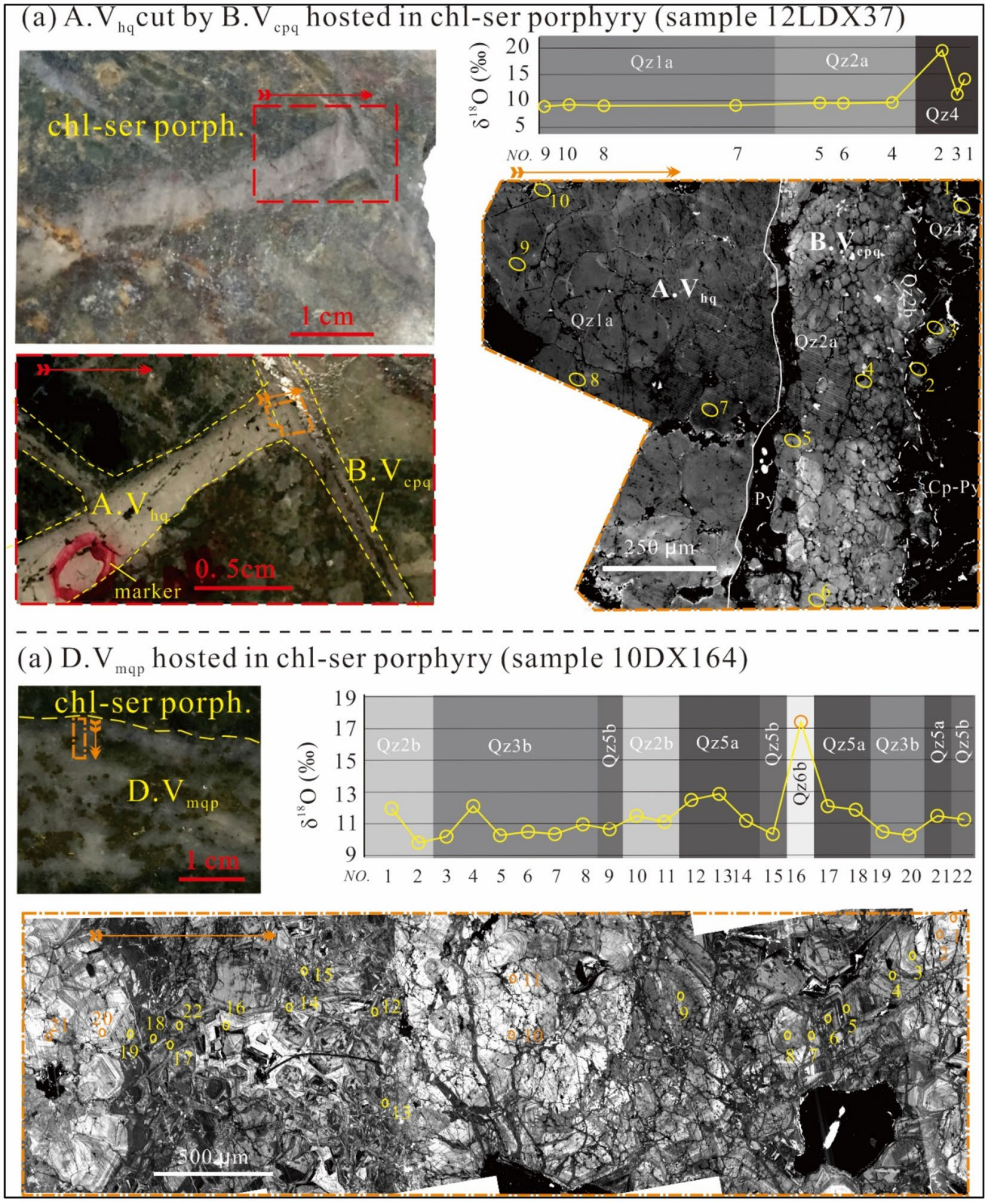
Cathodoluminescence microtextures and O isotopic analyses of A.V_aq_, B.V_cpq_, and D. V_mqp_ veins. (**a**) A A.V_hq_ vein was crosscut by a B.V_cpq_ vein in a chlorite-sericite altered porphyry. Qz1 and Qz2 had uniform δ^18^O values (9–10‰), whereas Qz4 had high values (up to 20‰) (the vein photographs and CL image were adapted from Liu et al.^26^); (**b**) A D.V_cpq_ vein hosted in chlorite-sericite porphyry. Qz2 was overprinted by Qz3, Qz5, and Qz6. Qz3 and Qz5 had slightly higher δ^18^O values (11–12‰), whereas Qz6 has much higher values (ca. 17‰). The ellipses were the analytical spots accompanied with spot numbers. The figure was reproduced using CorelDRAW2019 (http://www.coreldraw.com/en/).

**Figure 3:**
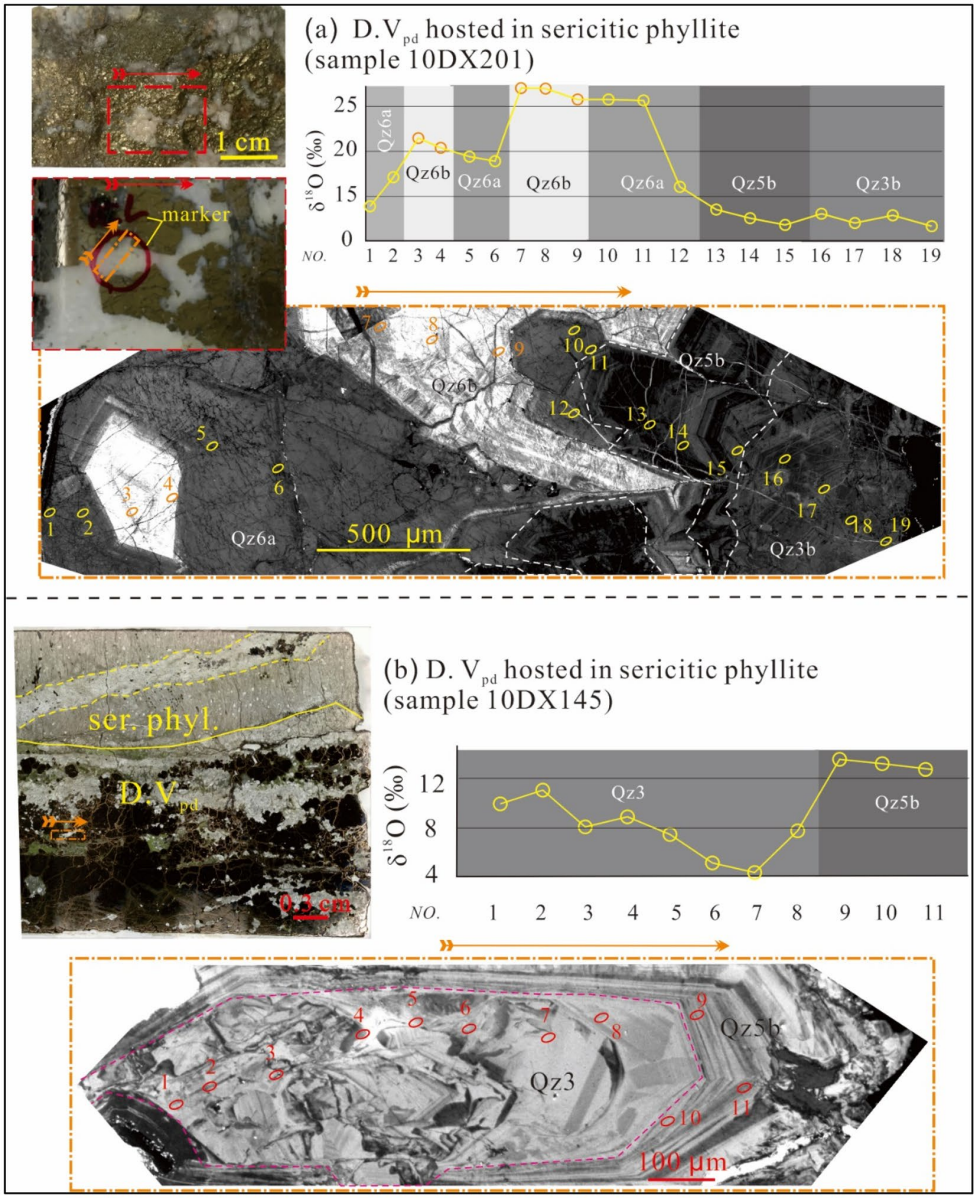
Cathodoluminescences microtextures and O isotopic analyses of D.V_pd_ veins hosted in a sericitic altered phyllite. (**a**) A profile traversing Qz3b, Qz5b, Qz6a and Qz6b revealed distinctive oxygen isotopic compositions (the CL image was adapted from Liu et al.^26^). (**b**) A quartz grain consisted of brecciated core (might be Qz3) and euhedral Qz5b overgrow (the vein photograph was adapted from Liu et al.^26^). The ellipses were the analytical spots with spot numbers. Noted that spots 6 & 7 in the Qz3 had low δ^18^O values (ca. 4‰). The figure was reproduced using CorelDRAW2019 (http://www.coreldraw.com/en/).

Detailed SEM-CL petrography by Liu et al.^26^ revealed six generations of quartz and four generations of anhydrite. Combining fluid inclusion and TitaniQ geothermometry, the authors also obtained formation temperatures for quartz. The CL textures and deposition temperatures are listed in the Table 2 and are briefly described below. The earliest quartz (Qz1) is characterized by mottled-CL texture (Fig. 2a) formed at 600–650 °C. The second quartz generation (Qz2) is characterized by an oscillatory core (Qz2a) and mottled rim (Qz2b), formed at 529–618 °C, and 473–551 °C, respectively. The third quartz generation (Qz3) commonly contains a dark interior (Qz3a) and oscillatory overgrowth (Qz3b) (Fig. 2b), and was formed at 374–392 °C and 350–450 °C, respectively. The fourth quartz generation (Qz4) is low in abundance and shows dark CL (Fig. 2a), and was formed at 310–390 °C. The fifth generation of quartz (Qz5) commonly consists of a dark core (Qz5a) and oscillatory overgrow (Qz5b) (Fig. 3a,b), which were formed at 356–360 °C and 344–351 °C, respectively. The latest quartz generation (Qz6) consists of dark to grey homogeneous domain (Qz6a) and very bright domain (Qz6b) (Fig. 3a), which were formed at 287–327 °C and 241–276 °C, respectively.

**Table 2:** A summary of generations, mineral assemblages, CL textures, and formation temperatures.

Generation	Cogenetic mineral	CL feature	Temperature (ºC)	Occurrence in sample
Qz1	Hematite	Mottled grey, “splatter and cobwebs”	600–650	12LDX37
Qz2a	Anh1a, K-feldspar, hematite, magnetite	Subhedral, oscillatory zoning, “splatter and cobwebs”	529–618	10DX22, 10DX172, 10DX328, 12DX500
Qz2b	Anh1b, hematite, magnetite	Mottled gry, “splatter and cobwebs”	473–551	10DX22, 10DX140, 10DX168, 10DX172, 10DX328, 12DX500
Qz3a	Anh2a, rutile, hematite, magnetite	Homogeneous dark	374–392	10DX140, 10DX168, 10DX220, 10DX328
Qz3b	Anh2b, epidote, rutile, pyrite, hematite, magnetite	Euhedral oscillatory zoning	350–450	
Qz4	Anh4, chlorite, sericite, rutile, bornite, chalcopyrite, pyrite, magnetite, monazite, apatite, epidote	Homogeneous, dark	310–390	10DX168, 10DX328, 12LDX37
Qz5a	Sericite, muscovite, pyrite, molybdenite, rutile, apatite	Homogeneous, dark	356–360	10DX164
Qz5b		Euhedral oscillatory zoning	344–351	10DX140, 10DX145, 10DX164, 10DX201, 10DX220
Qz6a		Euhedral oscillatory zoning, dark to grey	287–327	10DX140, 10DX201
Qz6b		Euhedral oscillatory zoning, very bright	241–276

**Figure 4:**
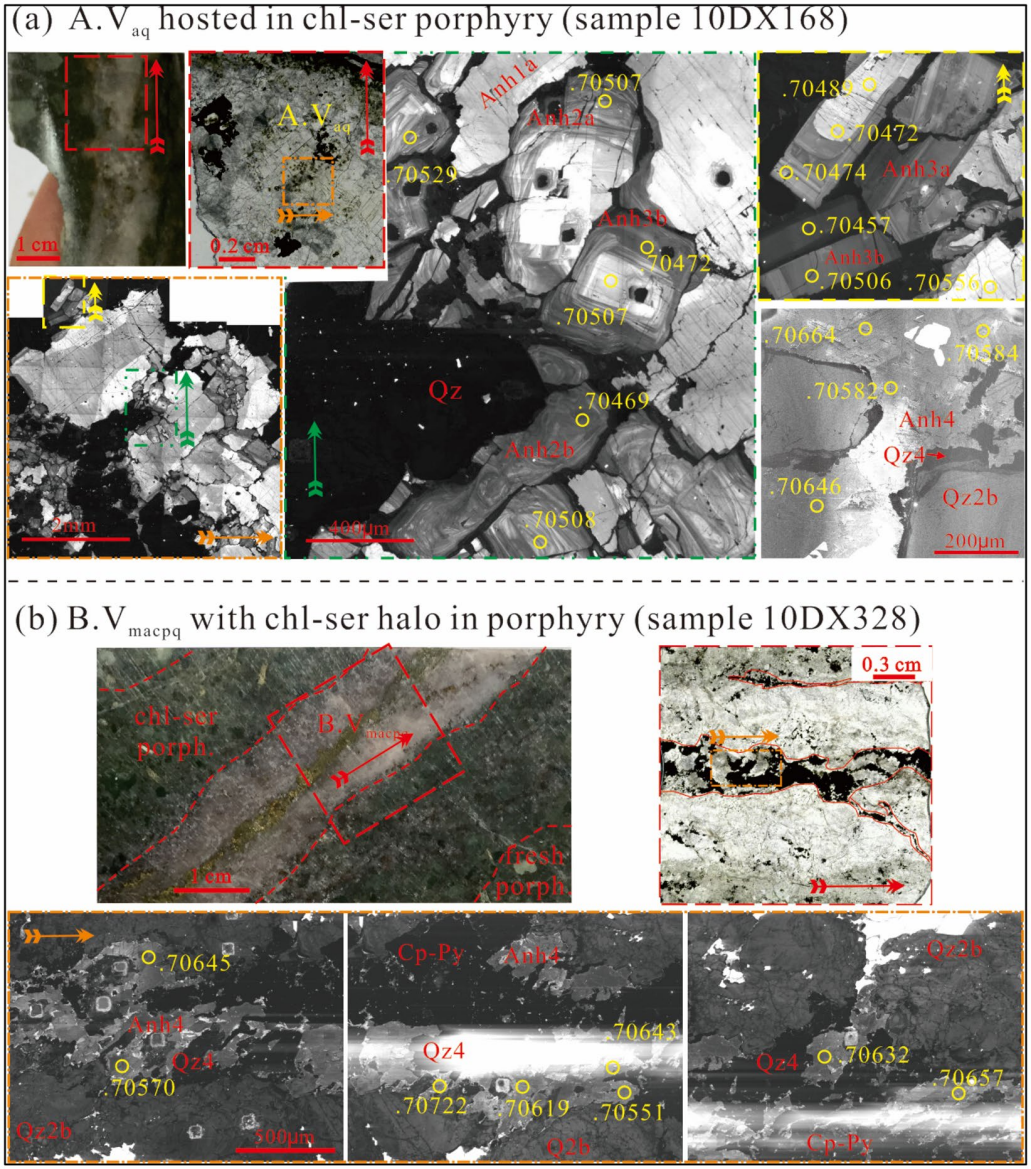
Cathodoluminescence microtextures and Sr isotopic analyses of anhydrite in A.V_aq_ and B.V_macpq_ veins in altered porphyry. (**a**) Four generations of anhydrite (Anh1 to Anh4) have distinctive CL and ^87^Sr/^86^Sr ratios (the vein photographs and CL images in the middle and upper right were adapted from Liu et al.^26^). Note that the Qz in the middle CL image contained two generations of quartz (Qz2 and Qz3), which is visible in Fig. 7d of Liu et al.^26^; (**b**) The anhydrite (Anh4) intergrown with quartz (Qz4), pyrite (Py) and chalcopyrite (Cpy) had high ^87^Sr/^86^Sr ratios (the vein photographs were adapted from Liu et al.^26^). Note that the overexposed strips in the CL images were caused by presence of tiny carbonates that had very high CL responses. Yellow cycles were laser spots accompanied with ^87^Sr/^86^Sr values. The figure was reproduced using CorelDRAW2019 (http://www.coreldraw.com/en/).

The first generation of anhydrite (Anh1) exhibits bright homogeneous to slightly patchy CL. It is subdivided to Anh1a and Anh1b according to CL intensity (Fig. 4a). The second generation (Anh2) has bright to grey CL with “wavy” oscillatory zoning (Fig. 4a). Anh2 is subdivided to brighter Anh2a and darker Anh2b. The third generation (Anh3) has euhedral morphology and display slightly oscillatory or homogeneous CL (Fig. 4a). The fourth generation (Anh4) is anhedral and CL-dark (Fig. 4b). All anhydrite types but Anh3 have been found in the A.V_aq_ and B.V_macpq_ and B.V_cpq_ veins due to repeated vein reopening^26^, whereas Anh3 occurs only in the A.V_aq_ vein in a minor amount.

The fluid history of Tongchang deposit has been divided into early (Qz1-Qz2-Anh1), early—intermediate (Qz3-Anh2), late—intermediate (Qz4-Anh4), and late (Qz5 and Qz6) stages based on quartz/anhydrite microtextures and deposition temperature^26^.

## Results

### Oxygen isotopic composition of quartz and fluid

**Figure 5:**
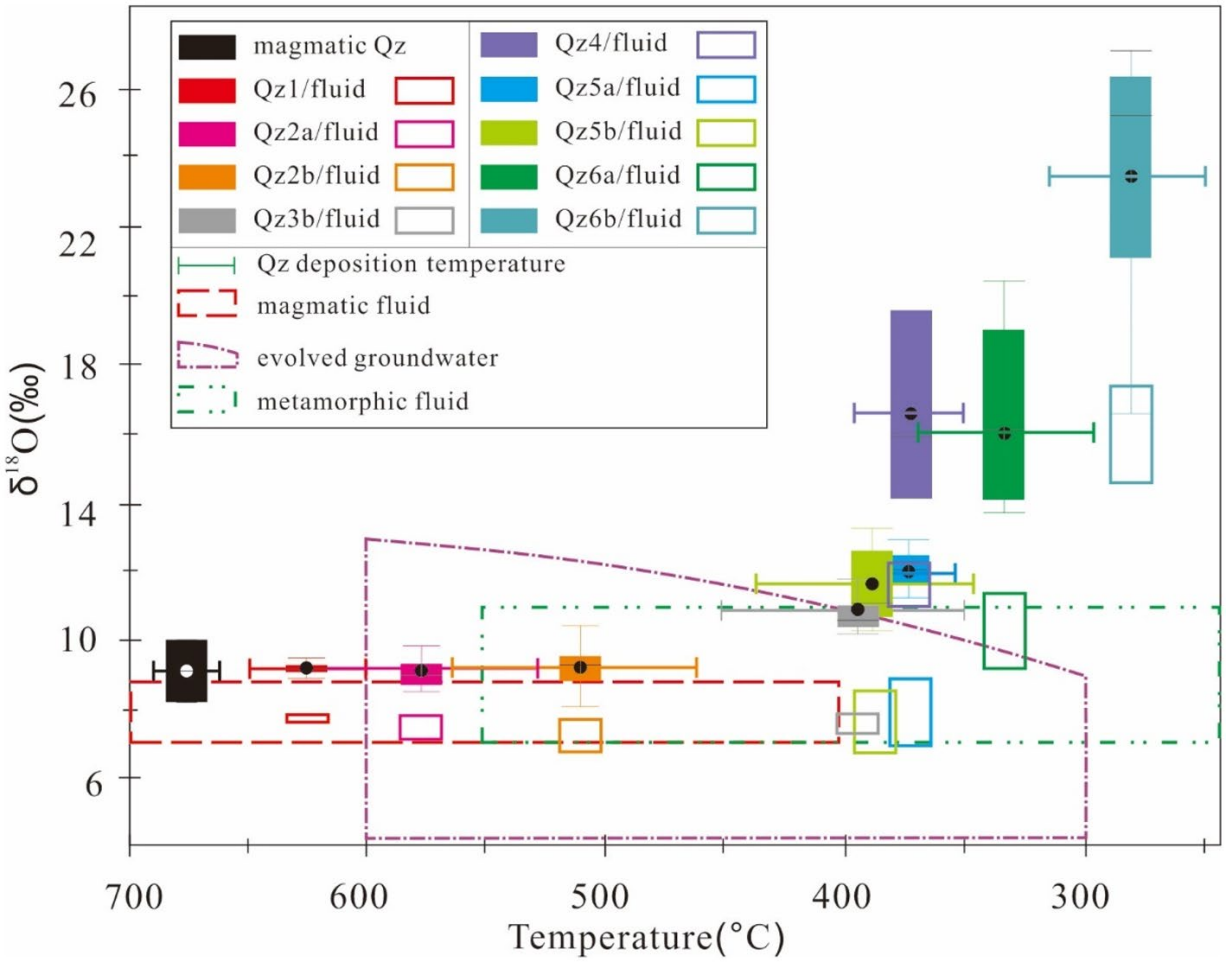
A Turkey boxplot of δ^18^O values for the Tongchang porphyry deposit. Magmatic fluid (7.0–8.8‰) is calculated from magmatic quartz (8.2–10.0‰^30^) and biotite (4.6‰^28^) with Ti-in-zircon temperature of the granodiorite porphyry^40^. Evolved groundwater and metamorphic fluid^41^ can also acquire magmatic signatures by interaction with phyllite. The figure was reproduced using CorelDRAW2019 (http://www.coreldraw.com/en/).

Oxygen isotope results are listed in the Table 3 and depicted in Figs. 2, 3, 5. Qz1 has clustered δ^18^O values of 9.0–9.3‰ (av. 9.2‰, N = 6). Qz2 shows a slightly larger δ^18^O range of 8.8–10.2‰ (av. 9.2‰, N = 20) for Qz2a, and 8.1–10.4‰ (av. 9.2‰, N = 44) for Qz2b. Qz3b has higher δ^18^O values of 10.2–13.0‰ (av. 10.9‰, N = 27). Qz4 exhibits distinctively high δ^18^O values of 14.1–19.6‰ (av. 16.6‰, N = 3); Qz5 displays a δ^18^O range of 11.2–12.9‰ (av. 12.0‰, N = 8) for Qz5a and 10.4–13.6‰ (av. 11.7‰, N = 17) for Qz5b. Qz6 peaks in δ^18^O at 13.9–20.4‰ (av. 16.5‰, N = 9) for Qz6a and 16.6–27.2‰ (av. 23.9‰, N = 16) for Qz6b. Above all, several low δ^18^O values (4.4–8.9‰) were detected in a relictic quartz in a phyllite-hosted D.V_pd_ vein (Fig. 3b).

**Table 3:** In-situ O isotopes of hydrothermal quart of the Tongchang porphyry Cu deposit.

Sample	δ^18^O_qz_ (‰)	2SE	δ^18^O_f_ (‰) min	δ^18^O_f_ (‰) max
QZ1				
12LDX37@7	9.2	0.3	7.6	7.9
12LDX37@8	9.1	0.2	7.5	7.8
12LDX37@9	9.0	0.3	7.5	7.8
12LDX37@10	9.3	0.3	7.8	8.0
12LDX37@11	9.1	0.2	7.6	7.9
12LDX37@12	9.3	0.3	7.7	8.0
Qz2a				
10DX22@1	8.9	0.2	6.8	7.4
10DX22@2	8.8	0.2	6.7	7.3
10DX22@3	8.8	0.2	6.8	7.4
10DX22@5	8.8	0.3	6.8	7.4
10DX22@6	9.4	0.2	7.3	7.9
10DX22@7	9.1	0.1	7.1	7.7
10DX22@8	8.9	0.2	6.9	7.5
12DX328@2	9.4	0.1	7.4	8.0
12DX328@3	9.0	0.2	6.9	7.5
12DX328@4	9.3	0.3	7.3	7.9
12DX328@5	9.0	0.2	7.0	7.6
12DX328@6	9.3	0.2	7.3	7.9
12DX328@7	8.9	0.3	6.9	7.5
12DX328@13	9.3	0.2	7.3	7.9
12DX328@14	8.9	0.2	6.8	7.4
10DX140@14	8.9	0.2	6.9	7.5
10DX140@20	10.2	0.4	8.2	8.8
12LDX37@4	9.8	0.2	7.8	8.4
12LDX37@5	9.7	0.3	7.7	8.3
12LDX37@6	9.7	0.2	7.6	8.2
QZ2b				
10DX22@4	9.3	0.3	6.7	7.4
10DX22@9	9.6	0.1	7.0	7.7
10DX22@10	9.8	0.2	7.3	8.0
10DX22@11	8.5	0.3	6.0	6.6
10DX22@12	9.2	0.1	6.7	7.4
10DX22@13	9.4	0.4	6.9	7.5
10DX22@14	9.3	0.3	6.7	7.4
10DX22@15	8.6	0.2	6.1	6.8
10DX22@16	9.3	0.3	6.7	7.4
10DX22@17	8.7	0.1	6.1	6.8
10DX22@18	8.8	0.2	6.3	6.9
10DX22@19	9.3	0.2	6.8	7.4
10DX22@20	9.2	0.2	6.7	7.4
10DX22@21	9.8	0.2	7.3	7.9
10DX22@22	8.8	0.3	6.3	7.0
10DX22@23	9.8	0.3	7.2	7.9
10DX22@25	9.4	0.1	6.8	7.5
10DX164@2	9.8	0.2	7.2	7.9
10DX172@1	9.0	0.5	6.5	7.2
10DX172@2	8.5	0.2	6.0	6.6
10DX172@3	8.8	0.2	6.3	6.9
10DX172@5	8.6	0.3	6.1	6.7
10DX172@6	9.0	0.3	6.5	7.1
10DX172@7	9.3	0.2	6.8	7.4
10DX172@8	8.7	0.2	6.1	6.8
10DX172@9	9.0	0.2	6.4	7.1
10DX172@10	9.5	0.3	6.9	7.6
10DX172@11	8.1	0.3	5.5	6.2
10DX172@12	9.1	0.2	6.5	7.2
10DX172@13	10.4	0.2	7.9	8.5
10DX172@14	8.5	0.2	6.0	6.6
10DX172@15	8.6	0.3	6.0	6.7
10DX172@16	8.9	0.1	6.4	7.0
10DX172@17	8.3	0.1	5.7	6.4
10DX172@18	9.1	0.3	6.5	7.2
10DX172@19	8.7	0.2	6.1	6.8
12DX328@1	9.5	0.2	6.9	7.6
12DX328@8	10.1	0.2	7.5	8.2
12DX328@9	9.4	0.2	6.9	7.5
12DX328@10	9.5	0.2	7.0	7.6
12DX328@11	9.4	0.2	6.8	7.5
12DX328@12	9.5	0.2	6.9	7.6
12DX328@15	9.8	0.2	7.3	7.9
12DX328@16	9.6	0.2	7.0	7.7
QZ3b				
10DX220@1	10.4	0.2	6.2	7.6
10DX220@2	10.5	0.2	6.4	7.7
10DX220@3	10.4	0.3	6.2	7.6
10DX220@4	10.2	0.3	6.1	7.4
10DX220@5	10.6	0.2	6.5	7.8
10DX220@6	10.9	0.2	6.7	8.1
10DX220@7	10.8	0.3	6.7	8.1
10DX220@8	10.5	0.1	6.3	7.7
10DX220@9	10.3	0.3	6.2	7.5
10DX220@10	10.7	0.1	6.5	7.9
10DX220@11	10.9	0.1	6.8	8.2
10DX220@12	10.4	0.2	6.3	7.7
10DX145@13	10.6	0.3	6.4	7.8
10DX140@9	11.2	0.2	7.1	8.4
10DX140@12	11.8	0.2	7.6	9.0
10DX164@3	10.2	0.2	6.0	7.4
10DX164@5	10.3	0.3	6.1	7.5
10DX164@6	10.5	0.3	6.3	7.7
10DX164@7	10.3	0.3	6.2	7.5
10DX164@8	10.9	0.1	6.8	8.2
10DX164@19	10.5	0.2	6.3	7.7
10DX164@20	10.2	0.3	6.1	7.5
10DX201@16	13.0	0.2	8.9	10.3
10DX201@17	12.0	0.2	7.9	9.3
10DX201@18	12.9	0.2	8.7	10.1
10DX201@19	11.7	0.2	7.6	9.0
QZ4				
12LDX37@1	14.1	0.2	7.6	9.9
12LDX37@2	19.6	0.1	13.0	15.3
10DX22@24	16.0	0.3	9.5	11.7
QZ5a				
10DX140@8	11.8	0.2	6.6	6.7
10DX164@4	12.1	0.2	6.9	7.0
10DX164@12	12.5	0.1	7.4	7.5
10DX164@13	12.9	0.2	7.8	7.8
10DX164@14	11.2	0.2	6.1	6.2
10DX164@17	12.1	0.2	7.0	7.1
10DX164@18	11.9	0.5	6.8	6.9
10DX164@21	11.5	0.3	6.4	6.5
QZ5b				
10DX145@9	13.6	0.3	8.1	8.3
10DX145@10	13.2	0.4	7.8	8.0
10DX145@11	12.8	0.3	7.3	7.5
10DX201@13	13.5	0.2	8.1	8.3
10DX201@14	12.6	0.4	7.1	7.3
10DX201@15	11.8	0.1	6.4	6.5
10DX140@1	11.7	0.3	6.2	6.4
10DX140@2	11.3	0.1	5.8	6.0
10DX140@3	12.2	0.2	6.8	7.0
10DX140@15	10.9	0.2	5.4	5.6
10DX140@16	10.6	0.2	5.2	5.4
10DX140@17	10.8	0.3	5.3	5.5
10DX140@18	11.3	0.3	5.8	6.0
10DX140@19	10.8	0.2	5.3	5.5
10DX140@21	10.4	0.3	5.0	5.2
10DX164@9	10.6	0.2	5.2	5.4
10DX164@22	11.2	0.3	5.8	5.9
QZ6a				
10DX201@1	13.9	0.2	6.6	7.9
10DX201@2	17.1	0.2	9.8	11.2
10DX201@4	20.4	0.2	13.1	14.4
10DX201@5	19.4	0.3	12.0	13.4
10DX201@6	18.9	0.2	11.5	12.9
10DX201@12	16.1	0.2	8.7	10.1
10DX201@22	14.8	0.2	7.5	8.8
10DX201@23	14.1	0.2	6.7	8.1
10DX201@28	14.2	0.3	6.8	8.2
QZ6b				
10DX201@3	21.5	0.3	12.1	13.7
10DX201@7	27.0	0.3	17.7	19.2
10DX201@8	27.0	0.4	17.6	19.2
10DX201@9	25.7	0.3	16.4	18.0
10DX201@10	25.8	0.2	16.4	18.0
10DX201@11	25.7	0.1	16.3	17.9
10DX201@20	24.7	0.3	15.4	16.9
10DX201@21	25.2	0.3	15.9	17.5
10DX201@24	26.9	0.2	17.6	19.2
10DX201@25	25.2	0.2	15.8	17.4
10DX201@26	25.5	0.2	16.2	17.7
10DX201@27	19.6	0.2	10.2	11.8
10DX164@16	17.4	0.3	8.1	9.7
10DX140@7	21.4	0.3	12.1	13.6
10DX140@11	27.2	0.2	17.8	19.4
10DX140@23	16.6	0.2	7.3	8.9

Oxygen isotope modeling suggests that hydrothermal fluids responsible for the early and early—intermediate stages (Qz1–Qz2 and Qz3) have δ^18^O values of 7 to 8‰ (Fig. 5). The late-intermediate stage fluid (Qz4) shows elevated δ^18^O (11–12‰). Qz5—precipitating fluid displays δ^18^O values similar to magmatic water and evolved groundwater (7–9‰). Qz6-depositing fluid exhibits high yet variable δ^18^O values (9.4–11.8‰ for Qz6a and 14.2–17.1‰ for Qz6b).

### Strontium isotopic composition of anhydrite

**Figure 6:**
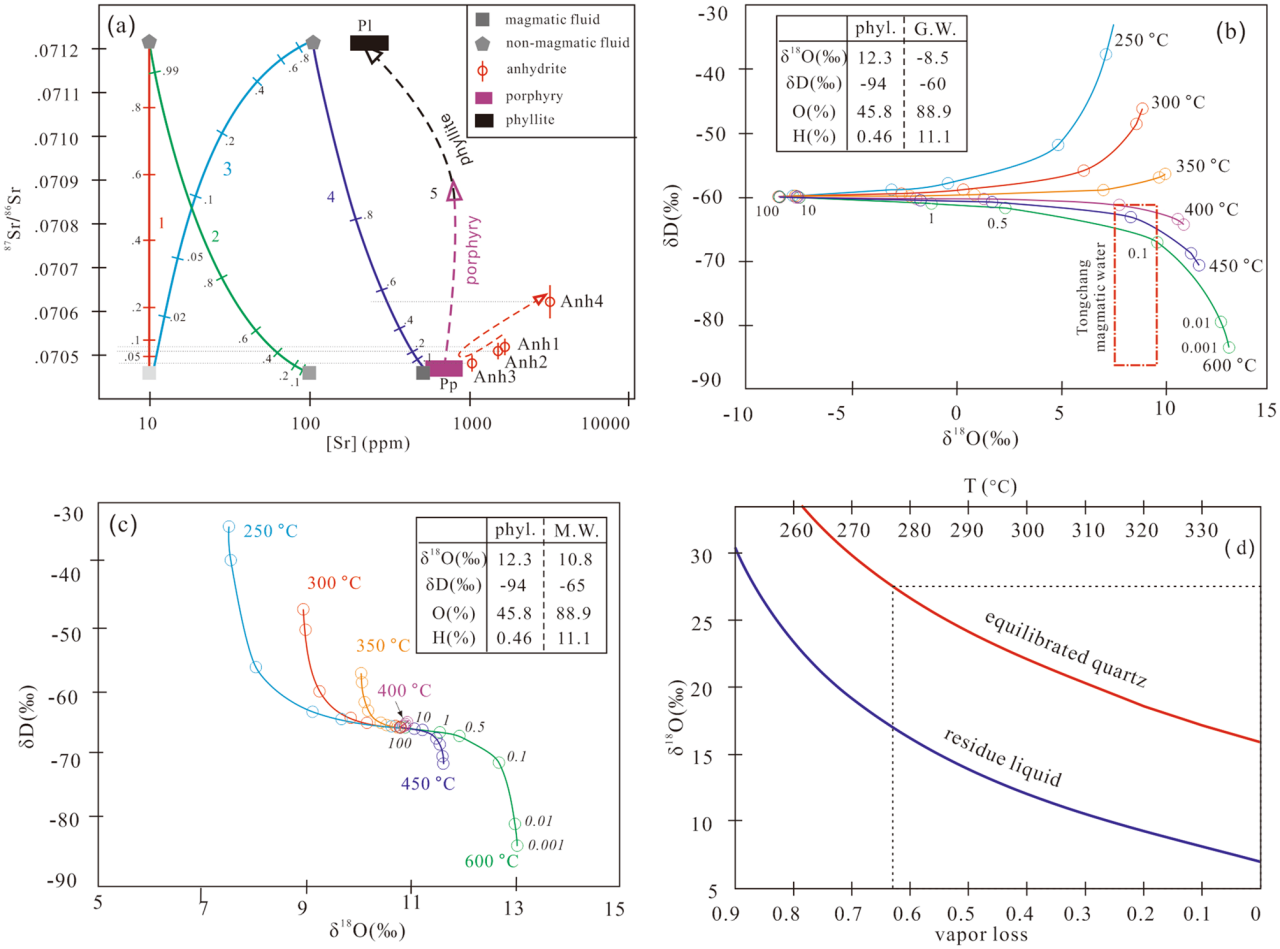
(**a**) In-situ Sr isotopic compositions of anhydrite (Anh1 to Anh4) compared with the porphyry (Pp)^35^ and phyllite (Pl)^27^. Also shown are modeled mixing curves between magmatic and non-magmatic fluids. (**b**) A fluid–rock interaction model for equilibrium fractionation between groundwater (G.W.) and phyllite. (**c**) A fluid–rock interaction model for equilibrium fractionation between metamorphic water (M.W.) and phyllite. (**d**) A Rayleigh distillation model showing O isotope fractionation during vapor loss between 340 and 250 °C. H–O isotope values of groundwater are from Zhang et al.^28^; metamorphic fluid values are from Zhao et al.^41^. The figure was reproduced using CorelDRAW2019 (http://www.coreldraw.com/en/).

Strontium isotope results are listed in Table 4 and depicted in Figs. 4 and 6a. Anh1, Anh2, and Anh3 have ^87^Sr/^86^Sr ratios of 0.70457–0.70527. A slight decreasing trend exists from Anh1 (0.70513) and Anh2 (0.70509) to Anh3 (0.70478). By contrast, Anh4 has evidently higher ^87^Sr/^86^Sr ratios (0.70583–0.70646). ^87^Rb/^86^Sr ratios for all analyzed spots are less than 0.001 and thus age correction is considered unnecessary. The measured ratios are considered representative of the anhydrite-depositing fluids.

**Table 4:** In-situ Sr isotopes of hydrothermal anhydrite of the Tongchang porphyry Cu deposit.

Spot no.	^87^Sr/^86^Sr	2σ	Spot No	^87^Sr/^86^Sr	2σ
Anh1a			12DX500 10	0.70518	0.00004
12DX500 01	0.70505	0.00005	12DX500 12	0.70507	0.00019
12DX500 08	0.70501	0.00005	12DX500 14	0.70509	0.00005
12DX500 09	0.70507	0.00005	12DX500 15	0.70512	0.00006
12DX500 13	0.70509	0.00004	12DX500 19	0.70512	0.00006
12DX500 21	0.70519	0.00005	Anh2b		
10DX168 33	0.70550	0.00005	12DX500 22	0.70506	0.00006
Anh1b			10DX168 02	0.70469	0.00005
12DX500 02	0.70499	0.00006	10DX168 03	0.70541	0.00005
12DX500 04	0.70510	0.00004	10DX168 05	0.70563	0.00005
12DX500 05	0.70516	0.00005	10DX168 06	0.70502	0.00004
12DX500 16	0.70500	0.00006	10DX168 20	0.70529	0.00005
12DX500 20	0.70505	0.00005	10DX168 21	0.70507	0.00007
10DX168 01	0.70513	0.00005	10DX168 24	0.70469	0.00006
10DX168 07	0.70510	0.00004	Anh3a		
10DX168 17	0.70556	0.00005	10DX168 10	0.70487	0.00005
10DX168 18	0.70473	0.00007	10DX168 12	0.70489	0.00005
10DX168 27	0.70523	0.00004	10DX168 13	0.70472	0.00006
10DX168 28	0.70528	0.00005	10DX168 14	0.70474	0.00005
Anh2a			10DX168 16	0.70506	0.00009
12DX500 03	0.70500	0.00006	10DX168 34	0.70467	0.00006
12DX500 07	0.70505	0.00005	10DX168 35	0.70475	0.00006
12DX500 11	0.70500	0.00004	10DX168 40	0.70485	0.00004
12DX500 17	0.70511	0.00006	Anh3b		
12DX500 18	0.70552	0.00006	10DX168 15	0.70457	0.00005
10DX168 08	0.70488	0.00005	10DX168 22	0.70472	0.00006
10DX168 23	0.70507	0.00004	Anh4		
10DX168 25	0.70508	0.00006	10DX328 01	0.70722	0.00016
10DX168 29	0.70494	0.00005	10DX328 02	0.70643	0.00003
10DX168 31	0.70512	0.00005	10DX328 03	0.70551	0.00003
10DX168 32	0.70505	0.00006	10DX328 04	0.70641	0.00004
Anh2b			10DX328 05	0.70632	0.00003
12DX500 06	0.70508	0.00005	10DX328 06	0.70657	0.00012
10DX328 07	0.70618	0.00005	10DX168 11	0.70593	0.00006
10DX328 08	0.70570	0.00013	10DX168 36	0.70664	0.00004
10DX328 09	0.70619	0.00004	10DX168 37	0.70582	0.00005
10DX328 10	0.70645	0.00009	10DX168 38	0.70646	0.00004
10DX168 04	0.70598	0.00009	10DX168 39	0.70584	0.00004
10DX168 09	0.70531	0.00005

## Discussion

### Fluid sources

There were possibly three types of hydrothermal fluids in the Tongchang deposit based on previous H–O isotope studies. Bulk H–O isotopic analyses revealed the presence of magmatic fluid and groundwater, with the former one having δ^18^O values of 7.0–8.8‰ and ^87^Sr/^86^Sr of 0.70455^29,30,35^, whereas the latter one having a δ^18^O value of − 8.2‰^27,28^. In-situ O isotope analyses of this study also revealed a ^18^O-depleted Qz3 (δ^18^O = 4.4‰, Fig. 3b), likely indicating precipitation from the groundwater that was circulating in the phyllite and slate. The host terrain was metamorphosed at greenschist conditions^34^, and the resultant metamorphic fluids had δ^18^O values of 10.4–11.2‰^41^.

The isotopic composition of these preexisting fluids can be modified by interacting with the host rock. Mass balance calculation suggests that groundwater can become ^18^O-enriched at a temperature of 600 °C and water/rock (w/r) ratio of 0.001(δ^18^O up to 13.0‰, Fig. 6b). Sr isotopes of the groundwater can approach that of the phyllite (^87^Sr/^86^Sr_(170 Ma)_ = 0.71215)^22^. Similar w/r reactions may have occurred for the metamorphic fluid between the timing of metamorphism (ca. 800 Ma) and ore formation (ca. 170 Ma). At temperatures above 300 °C, the metamorphic fluids can become^18^O-enriched and D-depleted, for instance, reaching a δ^18^O value of 13.0‰ at 600 °C and w/r of 0.01 (Fig. 6c).

### Pinpointing non-magmatic components

Paragenetic relation and O–Sr isotope analyses suggest that the early and early—intermediate fluids have a common fluid source, pointing to a mixture of magmatic and non-magmatic fluids. The relative proportion of the magmatic and non-magmatic end-members depends on Sr content (denoted as [Sr] thereafter) of the two fluids.

For the early-, early-intermediate stage, 10 ppm [Sr] is assumed for the non-magmatic fluids according to an overview of global metamorphic fluids by Wagner et al.^42^. The same value is assumed for the magmatic fluids based on studies of the Butte and Bajo de la Alumbrera porphyry deposits^43,44^. A binary mixing model suggests that Anh1, Anh2 and Anh3 predominantly comprise magmatic Sr (92%, 93% and 97%, respectively), and equivalently, 3% to 8% non-magmatic Sr (curve 1 in the Fig. 6a). Higher non-magmatic [Sr] values decrease the non-magmatic proportion (curve 2 in the Fig. 6a), whereas higher magmatic [Sr] values up to 100 ppm increase the non-magmatic proportion to ca. 45% (curve 3 in the Fig. 6a).

For the late-intermediate stage (Qz4-Anh4), using [Sr] of magmatic fluid of the Butte porphyry deposit (ca. 480 ppm^43^) and 100 ppm [Sr] for non-magmatic fluid^45^, calculation suggests 43% of magmatic and 57% of non-magmatic component (curve 4 in the Fig. 6a). This estimation, if correct, would indicate a highly-evolved groundwater and/or metamorphic water (δ^18^O = 12‰) that requires w/r interaction at low ratios (< 0.01). A fluid process that is capable of generating this fluid is similar to one developed for the Bingham porphyry Cu deposit^16^. In that model, the authors recognized a deep exchange zone where small patches of groundwater became ^18^O-enriched at high temperature and low w/r. These fluids were subsequently pumped to the deposition site at shallower levels where sulfide deposition and alteration occurred.

The Qz5—precipitating fluids were most likely the evolved groundwater and metamorphic fluid according to the O isotope results (Fig. 5). This inference is also supported by other observations First, bulk-rock Sr isotope analyses suggest that sericitic altered porphyry have high ^87^Sr/^86^Sr (up to 0.709), indicating a dominance of phyllite-derived Sr (curve 5 in the Fig. 6a). Second, the large volume of sericitic alteration requires large volumes of fluids that cannot be provided by magmas already cooled to low temperatures around 350 °C^46^.

The Qz6 exhibits high yet variable δ^18^O values with some being the highest in porphyry deposits (up to 27‰), corresponding to a parent fluid with high and variable δ^18^O values (from 9.4 to 17.1‰). This large spread in δ^18^O value is possibly due to Rayleigh distillation. It is worth noting that quartz Qz6a contacting directly Qz5 shows δ^18^O values close to that of Qz5 (Fig. 3a), likely indicating that Qz5 and Qz6a precipitated from the same fluid. Assuming the isotopic composition of Qz5-deposit fluid as the starting composition for the Qz6-depositing fluid, Rayleigh distillation modeling can produce the O isotope signatures in the Qz6a and Qz6b by cooling from 250 to 205 °C and vapor loss up to 70% (Fig. 6d).

### Mechanisms of non-magmatic fluid incursion

The Sr mixing calculation revealed a decreasing trend in the proportion of non-magmatic components (from 8 to 3%) between the early and early-intermediate stages. The decreasing trend is compatible with a scenario where non-magmatic fluids are progressively consumed in a closed system. Formation mechanism of a closed system in porphyry environments was studied by Fournier^3^. The author demonstrated the formation of an impermeable shell in the host rock through retrograde precipitation of quartz surrounding the porphyry intrusion at temperatures between 400 and 350 °C. The heat dissipated from the magmas also changes the rheology and thus hydrology of the host rock, forming a brittle to ductile transition interface (BDT) separating an internal ductile/lithostatic zone from an external brittle/hydrostatic zone. Interestingly, the BDT interface commonly coincides with the impermeable shell, and thus separates the internal magmatic fluids from the external groundwater.

**Figure 7:**
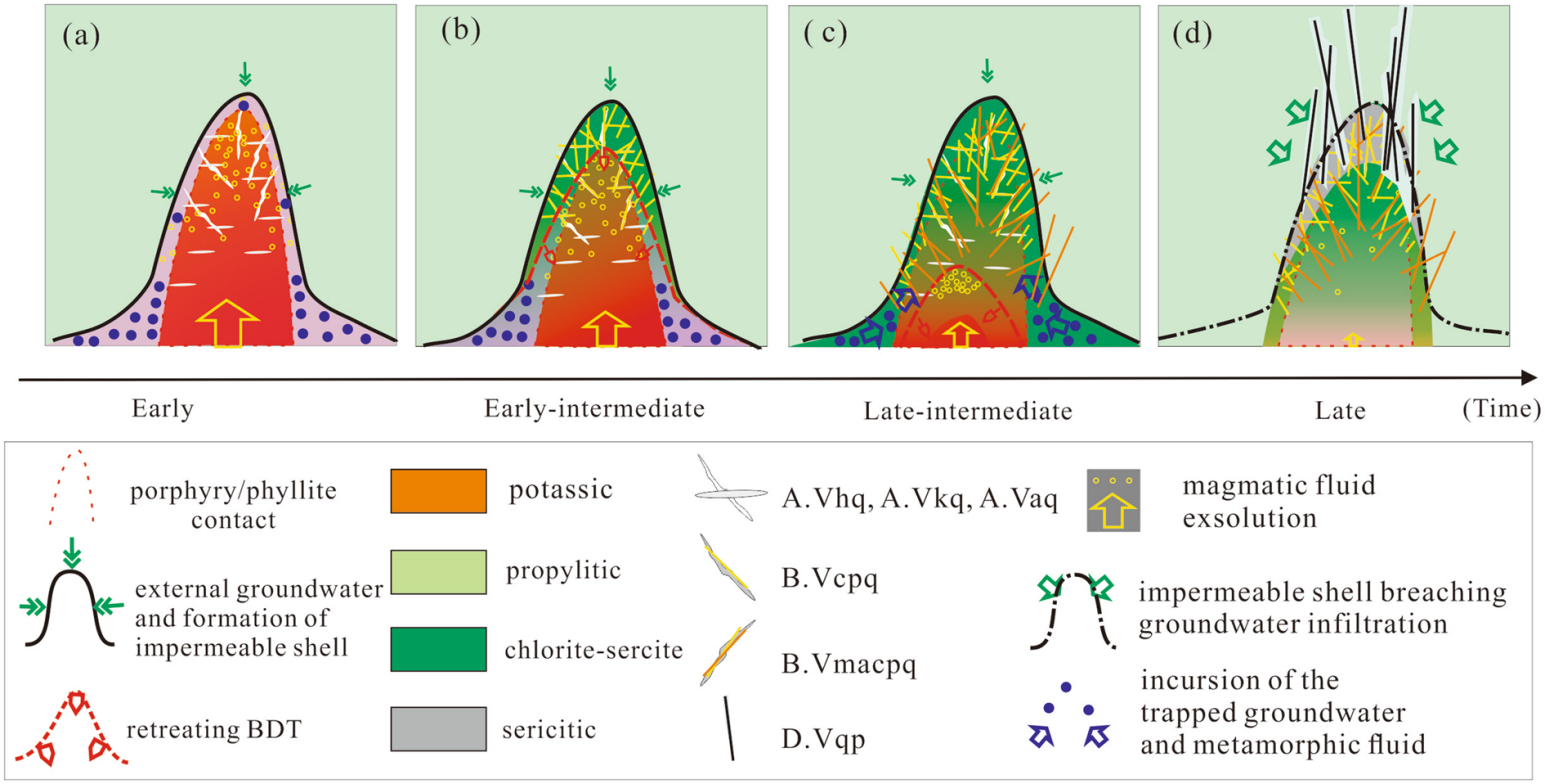
A genetic model for the formation of an impermeable shell and brittle/ductile transition (BDT) interface in the Tongchang porphyry Cu deposit. (**a**) In the early stage, retrograde precipitation of quartz formed a hydrologically impermeable shell, coinciding in space with the BDT interface. Non-magmatic components were trapped within the ductile phyllite. Disseminated chalcopyrite was formed by magmatic fluid reacting with mafic minerals in the porphyry. Early barren quartz veins were formed in fractures. (**b**) In the early-intermediate stage, the BDT moved downward, leaving behind a hydrostatic intermediate zone, which was ruptured upon fluid overpressures and formation of intermediate-temperature disseminated ores. (**c**) In the late-intermediate stage, the trapped non-magmatic components migrated upward and mixed with magmatic fluids, forming vein-type Cu sulfides. (**d**) Magmatic fluid production came to an end and induced external groundwater infiltration and ore remobilization. The figure was reproduced using CorelDRAW2019 (http://www.coreldraw.com/en/).

Nevertheless, the Fournier model did not consider possible entrainment of residual metamorphic fluid in the internal zone, and thus cannot explain the high δ^18^O signatures of the late-intermediate stage of the Tongchang deposit. Additionally, the Fournier model was built on multi-intrusion systems, where heat dissipation took place cyclically as opposed to a monotonic cooling in the mono-intrusion Tongchang deposit. In a mono-intrusion system, monotonic cooling would cause the BDT interface to migrate downward continuously and, therefore, create an intermediate region between the impermeable zone and the retreated BDT (Fig. 7a,b). This intermediate region is similar to the so-called “carapace” in many other orthomagmatic models^47^. The carapace zone can be repeatedly ruptured due to overpressures from the underlying cupola.

The late-intermediate stage at Tongchang may have undergone a major rupturing of the intermediate region as evidenced by the brecciation in Anh4, while the impermeable shell remained intact. Rupturing of the carapace zone may have released the enclosed non-magmatic components (Fig. 7c). Subsequently at the late stage, magmatic fluid production may have significantly decreased so that fluid pressure inside the magmatic—hydrothermal region was greatly reduced, causing volumetric contraction and breach of the impermeable shell. The breach may have induced invasive infiltration of the external groundwaters.

### Establishing and extending the Tongchang model

A genetic model is proposed for the Tongchang porphyry deposit based on the understanding of fluid reservoirs and spatiotemporal evolution (Fig. 7d). Similar to orthomagmatic models^47^, it commences with the establishment of a magmatic cupola, where magmatic fluids are accumulated. This is followed by the formation and downward migration of the impermeable shell and BDT interface.

The early and early-intermediate stages are dominated by magmatic fluids, which produced disseminated Cu sulfides through chemisorption reaction and potassic alteration^48^ (Fig. 7a), succeeded with vein-type Cu sulfides through fluid cooling and chlorite-sericite alteration (Fig. 7b). The late-intermediate stage was marked by incursion of non-magmatic fluids (metamorphic fluid) and subsequent mixing with magmatic fluid. The dilute and cool non-magmatic fluid caused chalcopyrite deposition due to rapid destabilization of ${CuCl}_{2}^{-}$ complexes^13^ and increase of ${H}_{2}S$ activity^2,3^ (Fig. 7c). The late stage was dominated by evolved groundwater, forming a high-sulfidation assemblage of pyrite and tennantite in the phyllic altered rocks (Fig. 7d). This is similar to the Butte porphyry deposit where deep protores (chalcopyrite-pyrite-magnetite) with potassic–chloritic alterations were remobilized by circulating oxidized groundwaters^49^.

**Figure 8:**
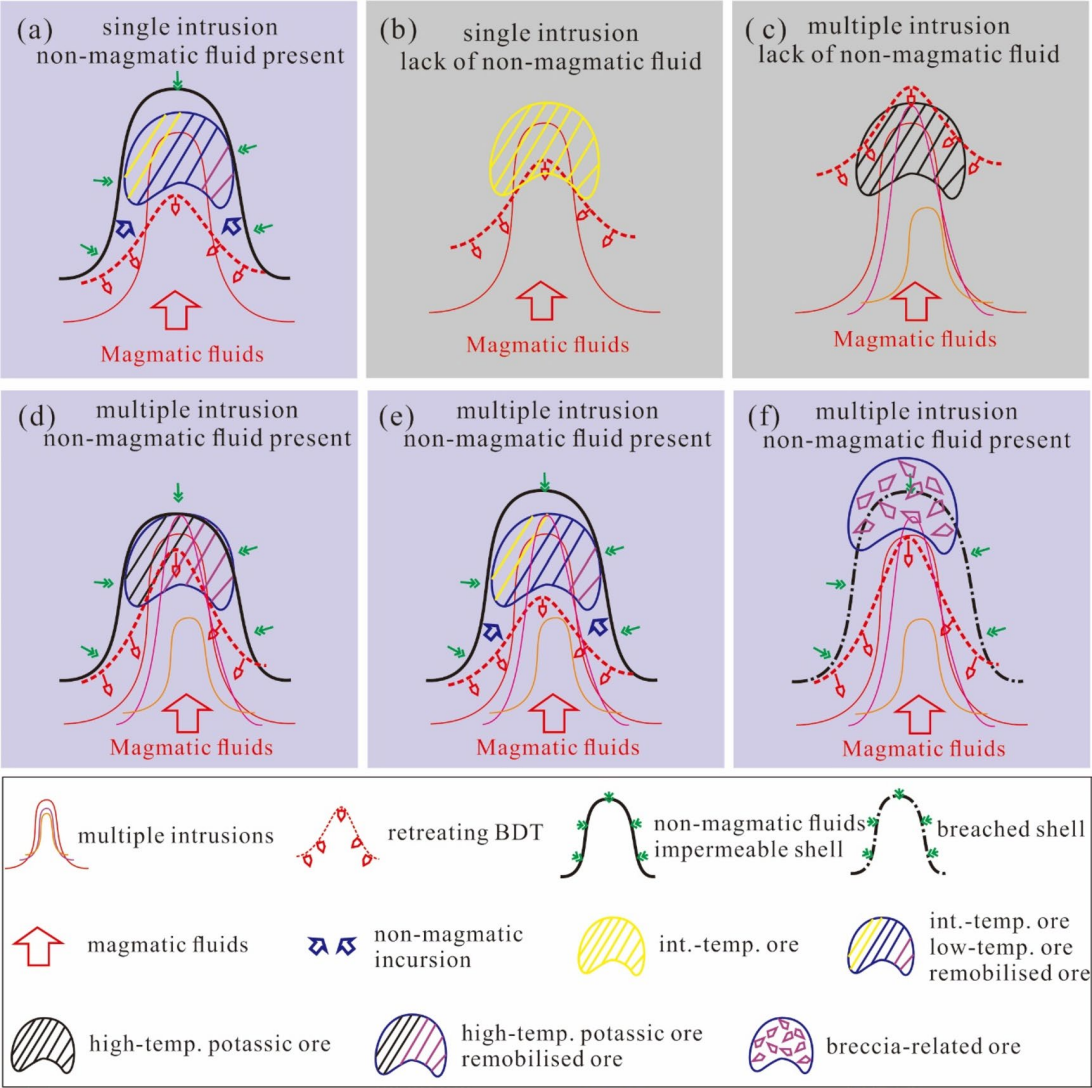
Six generic scenarios for porphyry ore formation, considering variabilities in non-magmatic availability (colored or grey background denoting with non-magmatic or without non-magmatic fluids) and intrusive history. (**a**) This scenario is the same as the Tongchang model. Ore style consists of intermediate-temperature ores, low-temperature ores, and remobilized ores. (**b**) and (**c**) represents mono- and multiple- intrusion system without non-magmatic components. The former will be dominated by intermediate-T ores, whereas the latter dominated by high-T ores. (**d**–**f**) represents multi-intrusion system with non-magmatic components and variable thermal histories depending on spatial configuration of intrusions. Ore style varies accordingly. The figure was reproduced using CorelDRAW2019 (http://www.coreldraw.com/en/).

The Tongchang model is further extended to account for variability in fluid availability of non-magmatic components and intrusive history (Fig. 8a). In porphyry deposits without non-magmatic fluids, such as those emplaced in contemporaneous volcanic and igneous rocks^50^, a thick impermeable shell cannot form due to a lack of external groundwater. Non-magmatic incursion would not happen, instead, such deposit would be dominated by magmatic fluids. Ore and alteration styles would resemble the early and early-intermediate stages of the Tongchang model, generating disseminated sulfides and chlorite-sericite alteration in the case of single intrusion (Fig. 8b), but potassic alteration in the case of multiple intrusions^49^ (Fig. 8c).

In multi-stock systems involving non-magmatic fluids, common in most porphyry deposits^5^, three scenarios are distinguished depending on the spatial configuration of the intrusions and the resulting thermal history. If repeated magma intrusion maintains the BDT interface at a location close to the impermeable shell until mineralizing fluid is completely consumed (Fig. 8d), little non-magmatic incursion would happen so that ores would be dominated by high-temperature disseminated sulfides. These ores would be remobilized by external groundwater at low temperatures if the impermeable shell was breached. In contrast, if repeated magma intrusion occurred in a way that allowed incursion of entrained non-magmatic components (Fig. 8e), ores would resemble that of Tongchang, for instance, in the El Salvador deposit the pre-mineral intrusions acted merely as host rocks^52^. If repeated magma intrusion caused the impermeable shell to breach at high temperatures, magmatic fluids would surge out and displace external groundwater circulation (Fig. 8f). This scenario similar to the Fournier model would result in phreatic brecciation of the country rocks, and led to breccia-related mineralization at depths and epithermal mineralization atop.

## Conclusion

Combined O and Sr isotope analyses provided new lines of evidence pointing to a transition from early magmatic-dominant to late non-magmatic dominant fluid system in the Tongchang mono-intrusion porphyry deposit. Non-magmatic components including isotopically evolved groundwater and metamorphic fluids were distinguished. The hydrological evolution of the deposit was largely controlled by dynamic interplay of the impermeable shell formed by retrograde mineral deposition and BDT interface. The initial non-magmatic incursion into the magmatic fluid promoted the major period of Cu sulfide deposition due to the diluting and cooling effects. The Tongchang model is applicable to mono-intrusion porphyry deposits where non-magmatic components are available. The Tongchang model is extended to six scenarios with broader implications.

## Methods

Nine selected quartz vein samples cover all vein and alteration types. Guided by SEM-CL petrography, quartz is analyzed for oxygen isotope with secondary ion mass spectrometry (SIMS) and anhydrite is analyzed for Sr isotopes with multi-collector LA-ICP-MS. Water–rock interaction and mixing models are constructed based on O–Sr isotope data.

### In-situ O isotope analyses

*In-situ* quartz oxygen isotopic compositions were measured using a Cameca IMS 1280 SIMS at the Institute of Geology and Geophysics, Chinese Academy of Sciences (IGGCAS). A Cs^+^ primary ion beam with an intensity of about 2 nA was accelerated at 10 kV and focused on an elliptic spot of ~ 10 × 20 µm in size. Oxygen isotopes were measured using multi-collection mode on two off-axis Faraday cups, with ^16^O counts being typically 109 cps during the analytical session. Obtained ^18^O/^16^O ratios were normalized to the VSMOW waters (^18^O/^16^O = 0.0020052), and then corrected for instrumental mass fractionation using NBS-28 (δ^18^O_VSMOW_ = 9.5‰^51^). A quartz standard (Qinghu, δ^18^O = 8.4 ± 0.2‰) was used to monitor instrumental drift. Measured δ^18^O values for Qinghu are between 8.3 and 9.1‰, with a mean of 8.6 ± 0.2 ‰ (1σ, n = 22). Analytical uncertainty for each analysis was normally better than 0.3–0.4‰ (2σ).

### In-situ Sr isotopes

Strontium isotopes in anhydrite were determined with a Neptune Plus MC-ICP-MS equipped with a MICRO/Las Pro 193 nm ArF excimer laser ablation system at IGGCAS. Laser parameters were set at a repetition rate of 8 Hz, a fluence of 10 J/cm^2^, and 90-μm spot size. Ion signals of ^83^Kr, ^84^Sr, ^85^Rb, ^86^Sr, ^87^Sr, and ^88^Sr were monitored by six Faraday cups at low-resolution mode. Prior to analysis, the ICP-MS was tuned to a maximum sensitivity using a standard solution. Analytical runs started with measurement of Kr gas for 40 s with laser-off, followed by laser ablation for 60 s. A piece of shark tooth (^87^Sr/^86^Sr = 0.709179 ± 0.000007, 2σ) as the primary standard, and Slyudyanka apatite (^87^Sr/^86^Sr = 0.70766‒0.70773) as the quality-check standard, were analyzed twice every ten unknowns^52^. Data reduction was conducted offline and potential isobaric interferences were resolved following the protocols described in Yang et al.^52^. Results of the Slyudyanka apatite suggested an analytical precision of ± 0.000113 (1σ) and accuracy of ± 0.000129.

### Oxygen and Sr isotope modeling

Oxygen isotopic compositions of fluids in equilibrium with quartz were calculated with the following equations ^51^:

$$\delta^{18} O_{qz} - \delta^{18} O_{fluid} = 2.05 \times \left( {\left( {T + 273.15} \right)^{2} } \right) - 1.14\;({5}00\;^\circ {\text{C }} < {\text{T}} < { 8}00\;^\circ {\text{C}})$$

$$\delta^{18} O_{qz} - \delta^{18} O_{fluid} = 3.34 \times \left( {10^{6} /\left( {T + 273.15} \right)^{2} } \right) - 3.31\; ({25}0\;^\circ {\text{C}} < {\text{T}} < {5}00\;^\circ {\text{C}})$$

Water–rock interaction models were derived using the following mass-balance equation (See the Tab “Water–rock interaction modeling” in the Appendix)^53^:

$${C}_{w}\times w\times {HO}_{H2O}^{i}+{C}_{r}\times r\times {HO}_{r}^{i}={C}_{w}\times w\times {HO}_{H2O}^{f}+{C}_{r}\times r\times {HO}_{r}^{f}$$

where $C$, $w$, $i$, $r$, $f$, and $HO$ stands for wt.% H or O, water, initial value, phyllite, final value, and δD or δ^18^O.

The H and O fractionation factors for phyllite (${\delta }^{18}{O}_{r}^{f}-{\delta }^{18}{O}_{H2O}^{i}$; $\delta {D}_{r}^{f}-\delta {D}_{H2O}^{f}$) were approximated by muscovite $2.68\times {10}^{6}\times {T (in K)}^{-2}-3.57$^54^, and $-22.1\times {10}^{6}\times {T \left(in K\right)}^{-2}+19.1$^54^, respectively.

Modeling of Sr mixing was based on the following equation (see the Tab “Sr mixing modeling” in the Appendix)^55^:

$${f}_{b}=\frac{{\left(\frac{{}^{87}Sr}{{}^{86}Sr}\right)}_{a}-{\left(\frac{{}^{87}Sr}{{}^{86}Sr}\right)}_{m}}{{\left[{}^{86}Sr\right]}_{b}/{\left[{}^{86}Sr\right]}_{a}\times \left({\left(\frac{{}^{87}Sr}{{}^{86}Sr}\right)}_{m}-{\left(\frac{{}^{87}Sr}{{}^{86}Sr}\right)}_{b}\right)+{\left(\frac{{}^{87}Sr}{{}^{86}Sr}\right)}_{a}-{\left(\frac{{}^{87}Sr}{{}^{86}Sr}\right)}_{m}}$$

where ƒ_*b*_ denotes weight percentage of non-magmatic fluid; subscripts *m*, *a* and *b* represent anhydrite, magmatic fluid, and non-magmatic fluid; [^86^Sr] denotes ^86^Sr concentration.

Rayleigh distillation fractionation modeling of the Qz6 fluid was based on the following equation (see the Tab “Rayleigh distillation modeling” in the Appendix):

$$R={R}_{0}\times {f}^{(\alpha -1)}$$

where $R$ denotes δ^18^O of the fluid, ${R}_{0}$ denotes δ^18^O of the starting fluid (8‰), $\alpha$ denotes fractionation factor (0.99).

### Supplementary Information

Supplementary Information.

## Data Availability

All data generated or analyzed during this study are included in this published article.
